# Recent applications of dioxinone derivatives for macrocyclic natural product and terpenoid synthesis

**DOI:** 10.3389/fchem.2022.1030541

**Published:** 2022-12-12

**Authors:** Kai Wei, Xinhua Zheng, Hongbin Zhang

**Affiliations:** ^1^ Henan Engineering Research Center of Funiu Mountain’s Medical Resources Utilization and Molecular Medicine, School of Medical Sciences, Pingdingshan University, Pingdingshan, Henan, China; ^2^ Key Laboratory of Medicinal Chemistry for Natural Resource, Ministry of Education, Yunnan Provincial Center for Research and Development of Natural Products, School of Chemical Science and Technology, Yunnan University, Kunming, Yunnan, China

**Keywords:** dioxianone, macrocyclic, macrolactam, terpenoid, macrolide

## Abstract

Dioxinone derivatives, a class of acetoacetate derivatives, have attracted widespread attention because of their multiple reactive sites, high reactivity, unique chemical properties, and potential synthetic applications. The dioxinone group is also stable under a wide range of reaction conditions, including strong acids, as well as a variety of transition-metal-catalysed processes, such as olefin metathesis and Pd-mediated cross-coupling. The inherent reactivity and diverse applications of dioxinones make them valuable reactive intermediates in organic synthesis. The conversion of dioxinones to acylketenes and their subsequent nucleophilic capture is also an excellent strategy for synthesising β-keto acid derivatives, which can be applied even in complex molecular synthesis. This review focuses on the recent advances in the application of dioxinones in synthetic method research and the total synthesis of natural products, highlighting the exceptional utility of these synthetic methodologies in the construction of macrocyclic cores and terpenoid skeletons. In particular, successful transformations of dioxinone fragments are discussed.

## Introduction

Chemistry has developed rapidly since its inception as a field of study in the 17th century. Chemists have made outstanding progress in the development of new reagents, reactions, and strategies for selectively and efficiently transforming organic compounds. These advances have been so profound that many highly complex natural products have been obtained through chemical synthesis. The development and utilisation of new reagents remain an area of intense interest in organic chemistry to maximise efficiency and practicability in total synthesis.

Among the numerous reagents used in organic chemistry, dioxinone derivatives have received considerable attention from organic chemists because of their multiple reactive sites, high reactivity, unique chemical properties, and potential synthetic applications. Moreover, dioxinone derivatives are increasingly being applied across a variety of research areas, including agrochemical development, natural product synthesis, and as chemical tools for a wide range of biological investigations.

Several factors have contributed to the popularity of dioxinone derivatives. Compound **1** is practical to use because it is inexpensive to prepare on a large scale using readily available commercial raw materials. Indeed, many chemical suppliers currently market **1** at reasonable prices ($ 290/kg for bulk quantities). More importantly, the steps used to synthesize various organic synthetic building blocks from **1** are typically robust, straightforward, and broad in scope.

**SCHEME 1:**
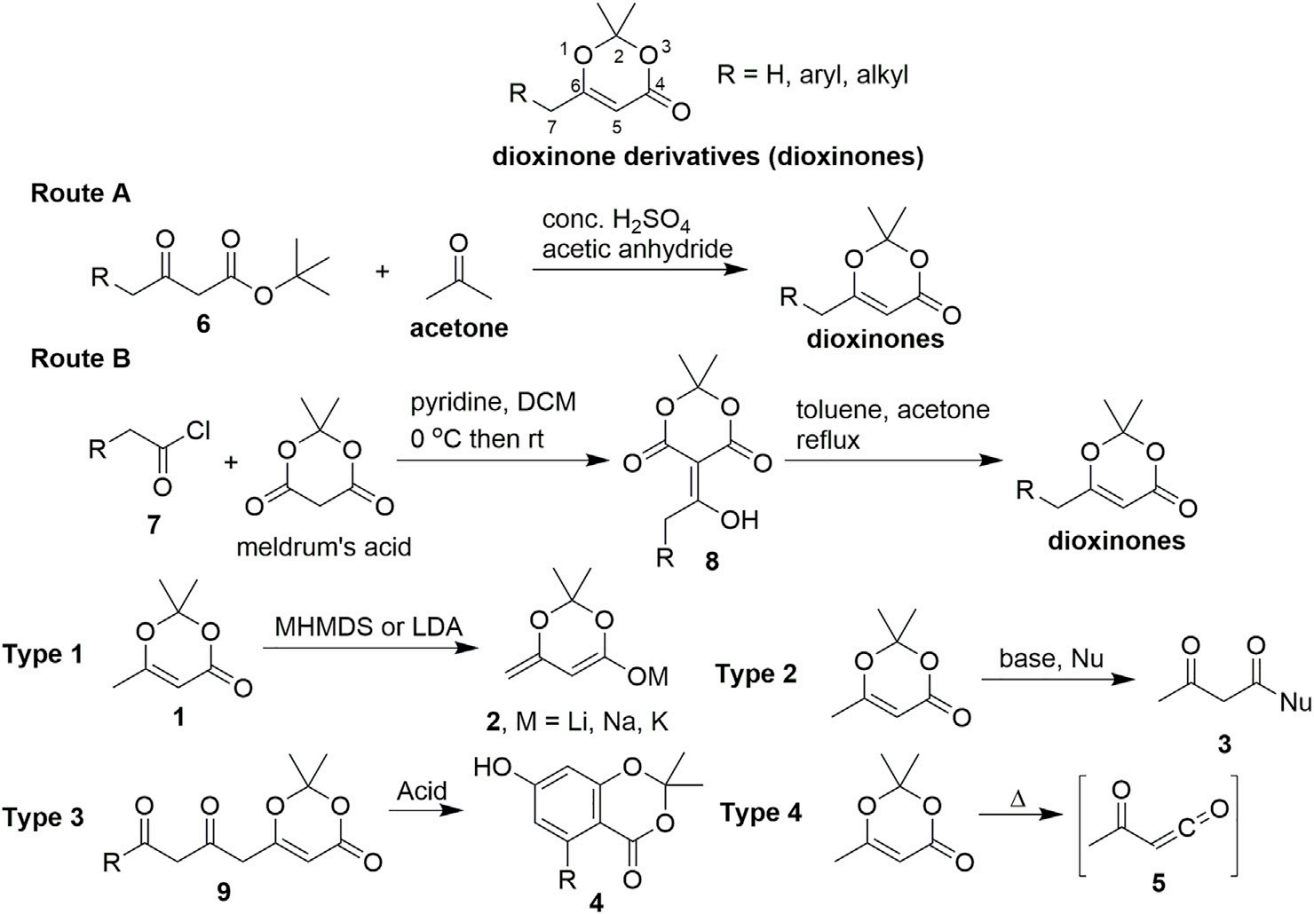
General sequence for the synthesis of dioxinone derivatives and their reactivity.

Various innovative methods have been developed for the preparation of dioxinone derivatives. Currently, two highly practical procedures (Scheme 1) are used by a large majority of chemical suppliers to produce **1**. The first method is a one-pot procedure using *tert*-butyl acetoacetate derivatives **6** as the starting materials in the presence of concentrated sulfuric acid, acetic anhydride, and acetone to obtain the dioxinone derivatives (route A) (Fuse et al., 2014). The other method uses meldrum’s acid and acid chloride **7** by a practical two-step procedure (route B) (Aoki et al., 2015).

Dioxinone derivatives first entered the purview of chemists as organic synthetic building blocks in the 1950s and have gradually become widely used. In 1989, Winkler reported the asymmetric synthesis of perhydrohistrionicotoxin (Winkler Harshberger, 1989), which was the first application of dioxinone in the total synthesis of a natural product. Dioxinone derivatives demonstrate multiple chemical properties because they can be considered a product of acetoacetate protected by acetone. There are several distinct types of this chemistry (using compound **1** as an example in Scheme 1): 1) The direct reaction of C-7 in **1** with a wide range of bases (e.g., MHMDS or LDA) proceeds to give **2** or enol silyl ethers (Scheme 1, type 1) in high yields. Kalesse reviewed the application of dioxinones in vinylogous aldol reactions in 2005 (Kalesse, 2005). 2) Compound **1** enables clean and high-yield additions of a very wide range of diverse nucleophiles, including organo-magnesium, lithium, and zinc reagents; stabilised carbanions exemplified by enolates; and numerous hydride reagents (Scheme 1, type 2). Compound **1** can also be converted to an acylketene reactive intermediate **5**) under high-temperature conditions, exhibiting rich chemistry (Scheme 1, type 4). Reaction types 2 and 4, which provide direct access to β-keto lactones and β-keto lactams, respectively, have been effectively utilised in complex, target-directed synthesis. Intermolecular or intramolecular trapping of reactive acylketenes by nucleophiles gives rise to valuable structures and enables the execution of challenging and delicate bond formations that might be difficult to achieve using alternative synthetic strategies. Sorensen reviewed the application of dioxinones to bond formation by intermolecular and intramolecular trappings of acylketenes in the total synthesis of large-ring natural products in 2009 (Reber et al., 2009). 3) Moreover, a cyclisation–aromatisation cascade process can be accomplished by an acid-catalysed reaction with the dioxinone derivatives **9**, which serves as an electron-rich reagent widely used in the synthesis of resorcylate natural products (Scheme 1, type 3).

This review aims to highlight recent strategic applications of dioxinone derivatives in natural product synthesis that were not covered in previous reviews (Kalesse, 2005; Reber et al., 2009), and emphasise the significant role they play in generating molecular skeletons. Only representative examples in which dioxinones are used as a crucial step in the construction of either the core structure or the key structural motif of the target molecule are presented. General applications of the selected examples of total synthesis are grouped based on the natural product types, including macrocyclic natural products, terpenoids, and some applications in research on synthetic methods, with a particular focus on studies from the last decade.

## Macrocyclic natural product synthesis

The most common application of acylketenes in organic synthesis is the preparation of β-keto acid derivatives for construction of macrocyclic natural product frameworks, which are widely used in the synthesis of macrolides and macrolactams. Boeckman and Pruitt were the first to use dioxinones as precursors to acylketenes in the synthesis of complex natural products featuring macrolactones and macrolactams, as reviewed by Sorensen (Reber et al., 2009). Here, we briefly introduce other studies. In this section, select recent examples are presented to illustrate contemporary solutions to problems involving dioxinones.

**SCHEME 2:**
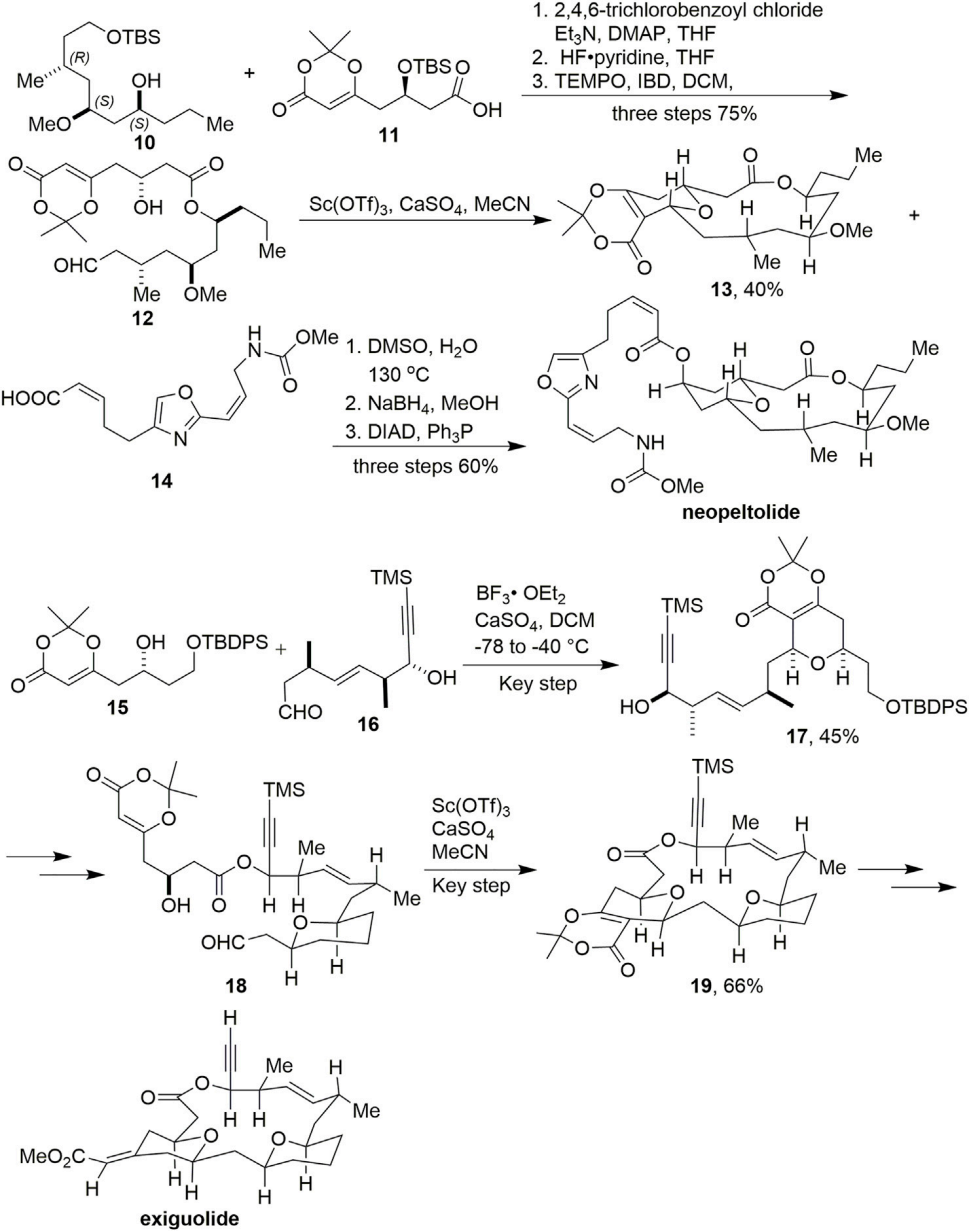
Scheidt’s synthesis of neopeltolide and exiguolide.

In 2009, Scheidt and coworkers reported the successful synthesis of neopeltolide (Scheme 2, Custar et al., 2008, 2009). Dioxinone **1** was used as an important functional block to construct intermediate **11** *via* a vinylogous aldol reaction. After condensation, deprotection of TBS, and oxidation, key intermediate **12** was obtained. Macrocyclic **13** was successfully acquired in 25% yield by cyclisation *via* an intramolecular Prins reaction promoted by Sc(OTf)_3_.

In 2011, This group constructed the exiguolide skeleton using the same strategy *via* two Prins cyclisations and ultimately completed the total synthesis of exiguolide (Scheme 2, Crane et al., 2011). The first Prins reaction was successfully mediated by BF_3_·Et_2_O with compounds **15** and **16** to produce **17** in 45% yield. After multiple transformations, the second key Prins reaction was promoted by Sc(OTf)_3_ to construct macrocyclic intermediate **19** in 66% yield.

**SCHEME 3:**
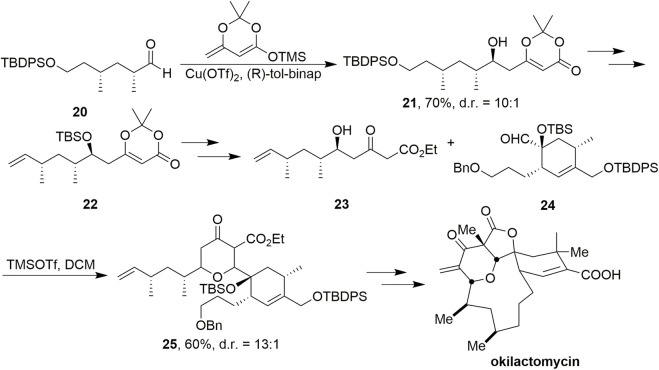
Scheidt’s synthesis of okilactomycin.

Dioxinone was also used as an important synthetic block to provide the skeleton in the synthesis of okilactomycin by Scheidts’ group (Scheme 3, Tenenbaum et al., 2011). Using copper-catalyzed vinylogous aldol reaction conditions with dioxinone silyl enol ether and aldehyde **20**, β-hydroxy dioxinone **21** was formed in 70% yield and 10:1 diastereomeric ratio favoring the desired product. After multistep transformations to obtain **22**, treatment of it with KOEt smoothly provided a β-ketoester, where the protecting group was removed with HFpy to afford **23** without any observed lactonization. The conditions of the stereoselective coupling of **23** and **24** were TMSOTf in DCM, and led to the desired **25** in 60% yield and as a 13:1 mixture of diastereomers favoring the desired 2,6-cis isomer. Ultimately, (–)-okilactomycin has been achieved successfully.

**SCHEME 4:**
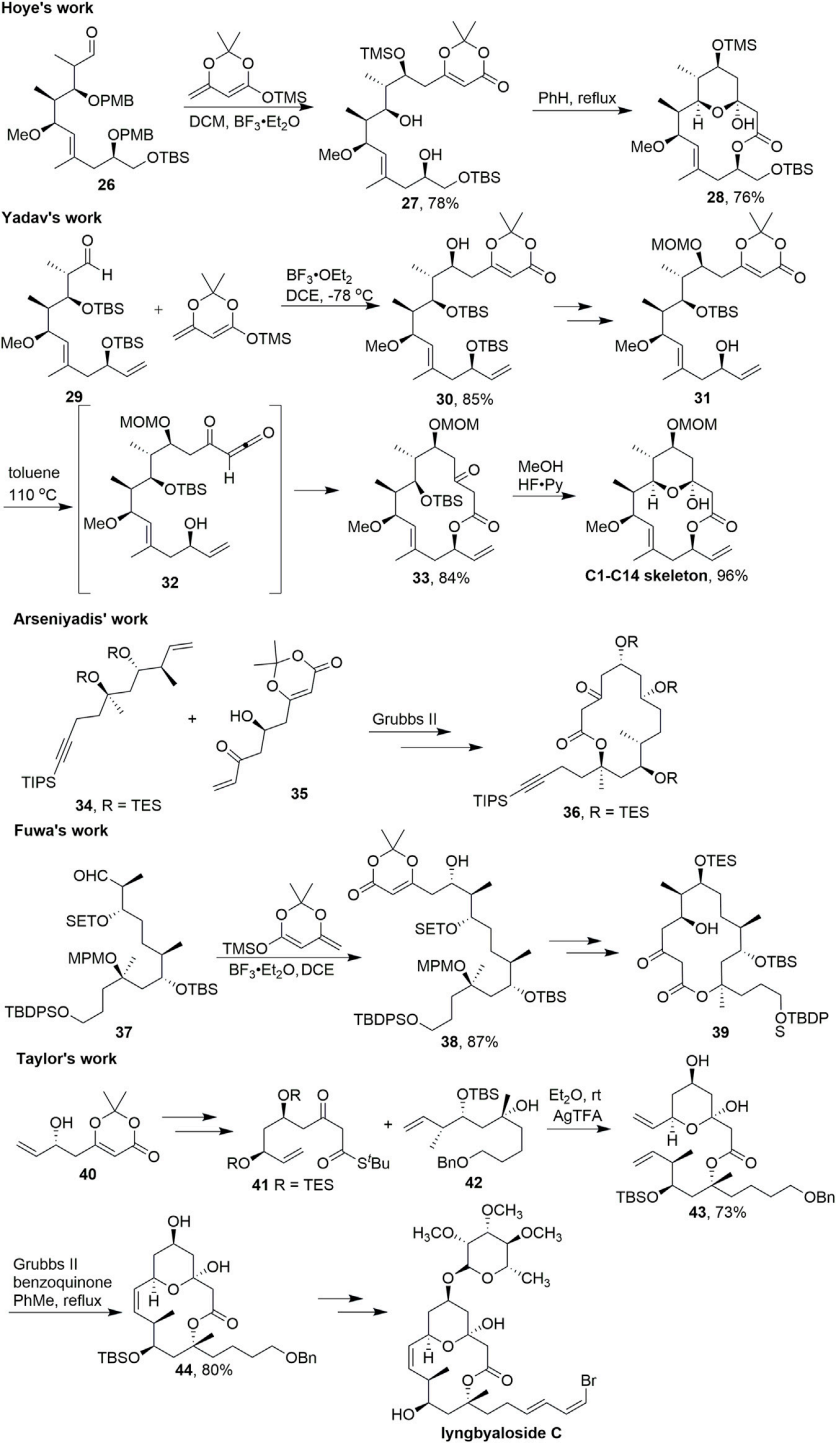
Synthesis of a 14-membered macrolactone central macrolide.

Callipeltoside is a popular target for total synthesis because of its complex architecture and promising anti-tumour bioactivity (Zampella et al., 1996). Like the related compounds lyngbouilloside and lyngbyaloside, these two natural products feature a 14-membered macrolactone with a transannular hemiketal. In 2010, Hoye et al. (2010) reported the asymmetric total synthesis of the macrolide natural product callipeltoside A (Scheme 4). After a vinylogous aldol reaction with **26**, acylketene macrolactonisation took place with a high degree of regioselectivity. Acylketene precursor **27** contains two unprotected hydroxyl groups, yet only the single constitutional isomer **28** was observed after heating this substrate in refluxing benzene.

In 2012, Yadav et al. (2012) used almost the same strategy to build the skeleton in the total synthesis of callipeltoside A (Scheme 4). By employing a diastereoselective aldol addition from the C5–C14 aldehyde segment **29** and dienyl silyl ether, afforded the adduct **30** as the only product in 85% yield. After several steps, compound **31** was received in good yield. Thus, refluxing of a dilute solution of **31** in toluene induced the loss of acetone through thermal decomposition to evolve the acylketene intermediate **32**, which was then trapped intramolecularly by the secondary hydroxy at C13 to generate the 14-membered lactone **33** in 84% yield. The final synthetic operation was carried out by using HF·Py to transform 33 to the C1–C14 skeleton of (–) callipeltoside A by removing the silyl group to form the requisite tetrahydropyran ring.

Similar strategies targeting the central macrolide were reported by Arseniyadis (ElMarrouni et al. 2011), Fuwa (Fuwa et al., 2015), and Taylor (Chang et al., 2015) (Scheme 4). Arseniyadis successfully used C–C bond formation *via* olefin metathesis with key intermediates **34** and **35**; **36** was obtained by heating the dioxinone intermediate. Vinylogous aldol reaction was used to construct the key intermediate **38** to accomplish the subsequent 14-membered macrocyclic **39** in Fuwa’s study. Taylor and coworkers prepared **41** from **40** to obtain key intermediate **43** using AgTFA; Then **43** served as the precursor for olefin metathesis. After the 14-membered macrocyclic **44** was obtained by olefin metathesis cyclization, lyngbyaloside C was synthesized multi-step functional group transformation.

**SCHEME 5:**
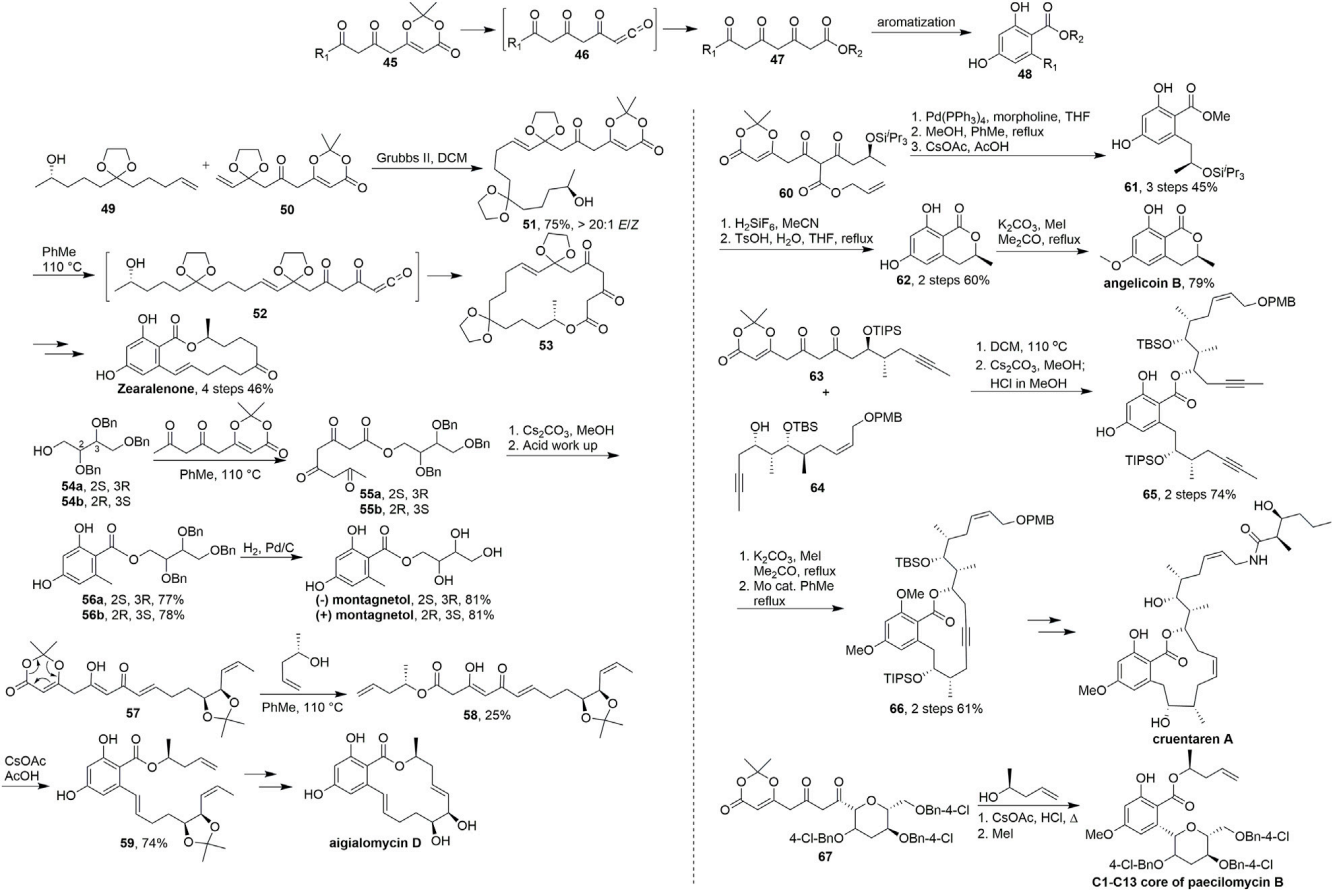
Barrett’s synthesis of resorcylate natural products.

In 2008, Barrett’s group developed a new route (Navarro et al., 2008) to resorcylate natural products which was inspired by biosynthetic considerations and was based on macrocyclisation and transannular aromatisation of the dioxinone fragment (Scheme 5). The lactones **48** containing these units utilizing tandem late stage aromatization was obtained from 2,4,6-triketo-ester precursors **47**, which was prepared with the dioxinone derivatives **45** by efficient trapping of the resultant ketene **46** with alcohols.

In their research (Miyatake-ondozabal and Barrett. 2010), key advanced intermediate **51** was obtained in 75% yield (E/Z > 20/1) *via* olefin metathesis using a Grubbs II catalyst. Then, **51** was converted into acyl ketene intermediate **52** under refluxing toluene, and intramolecular hydroxyl capture led to the formation of 18-membered macrocyclic lactone **53**. The synthesis was completed *via* multistep transformations and transannular aromatisation. Barrett et al. have used this strategy from 2010 to 2018 in some brilliant studies on the synthesis of resorcylate type natural products.

Thermolysis of dioxinone in the presence of benzyl-protected erithritols **54a** and **54b** gave the triketo-esters **55a** and **55b**, respectively. Cyclisation and aromatisation followed by hydrogenolysis of the benzyl groups gave (+)-montagnetol. (+)-Erythrin also could be prepared based on the same route in this work (Scheme 5, Basset et al., 2010). Upon heating in toluene, the dioxinones **57** was trapped with chiral alcohol to generate the ketene **58** and directly aromatized by reaction with cesium acetate followed by acetic acid to give the resorcylates **59** respectively in 74% overall yields. After the key RCM and deprotection of the acetonide moiety, aigialomycin D was achieved successfully (Scheme 5, Calo et al., 2009). In 2011, Barrett and coworkers (Scheme 5, Anderson et al., 2011) used one pot palladium(0)-catalyzed deallyation-decarboxylation-ketene trapping-aromatization to give the desired resorcylate **61** in 45% yield over three steps. Deprotection of the silyl ether and acid catalyzed cyclisation gave lactone **62** in 60% yield over two steps. Finally, regioselective methylation of **62** provided angelicoin B. The core resorcylate unit **65** of cruentaren A was synthesized by thermolysis of diketo-dioxinone **63** afforded the corresponding highly reactive ketene, which was trapped with **64** in 74% yield over two steps. After the ring closing by alkyne metathesis and methylation, the key intermediate **66** was obtained and finally cruentaren A was achieved in several steps from **66** (Scheme 5, Fouché et al., 2012). The C1 to C13 tetrahydropyranyl-resorcylate core of paecilomycin B was also synthesized by this strategy in 2018 (Scheme 5, Cookson et al., 2018).

**SCHEME 6:**
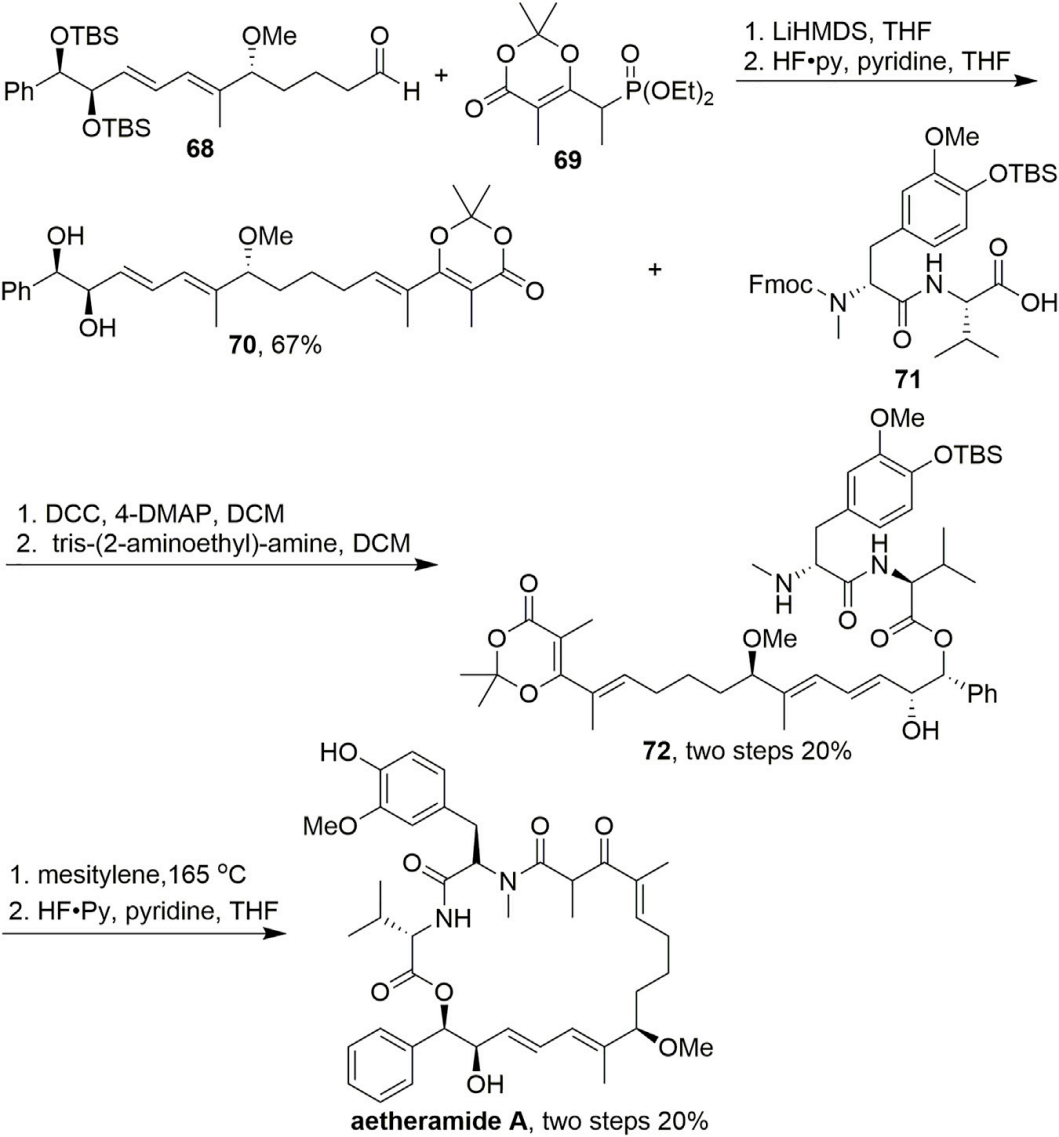
Kalesse’s synthesis of aetheramide A.

In addition to its usefulness for macrolide synthesis, dioxinone is also an excellent fragment for the nucleophilic addition of amines to form macrolactams. In 2016, Kalesse reported the synthesis of aetheramide A, which is a highly potent anti-HIV reagent (Scheme 6, Gerstmann and Kalesse, 2016). In their research, to access precursor **72**, dioxinone **69** was used as a Horner–Wadsworth–Emmons (HWE) resource in an HWE reaction. The dioxinone moiety was introduced through this olefination, and the final TBS deprotection step completed the synthesis of polyketidic fragment **70** in 67% yield. After condensation with acid **71**, mesitylene led to the formation of the acylketene intermediates, which were trapped intramolecularly by the secondary amine. Deprotection was then carried out using HF-pyridine to give aetheramide A. This synthesis is particularly interesting because although there are various well-established procedures for macrolactamisations using unsubstituted dioxinones, examples with dioxinones bearing a methyl group are rare, possibly because of the considerably higher temperatures necessary to initiate the retro Diels–Alder reaction.

Captures of acylketenes are particularly useful in a synthetic context because sometimes medium and large rings can be formed, which are often difficult to synthesise using more typical methods. The diverse reactivities and applications of dioxinones make them valuable reactive synthetic blocks for macrocyclic natural products. Many similar synthetic studies have been reported (Rentsch and Kalesse, 2012; Dalby et al., 2013; Ogura et al., 2016), which will not be repeated here, in addition to those described earlier.

## Terpenoid synthesis

Terpenoids are structurally intriguing natural products that have attracted extensive attention owing to their unique and complex structural characteristics and diverse biological activities. Dioxinone derivatives also play an important role in the total synthesis of some complex terpenoids.

**SCHEME 7:**
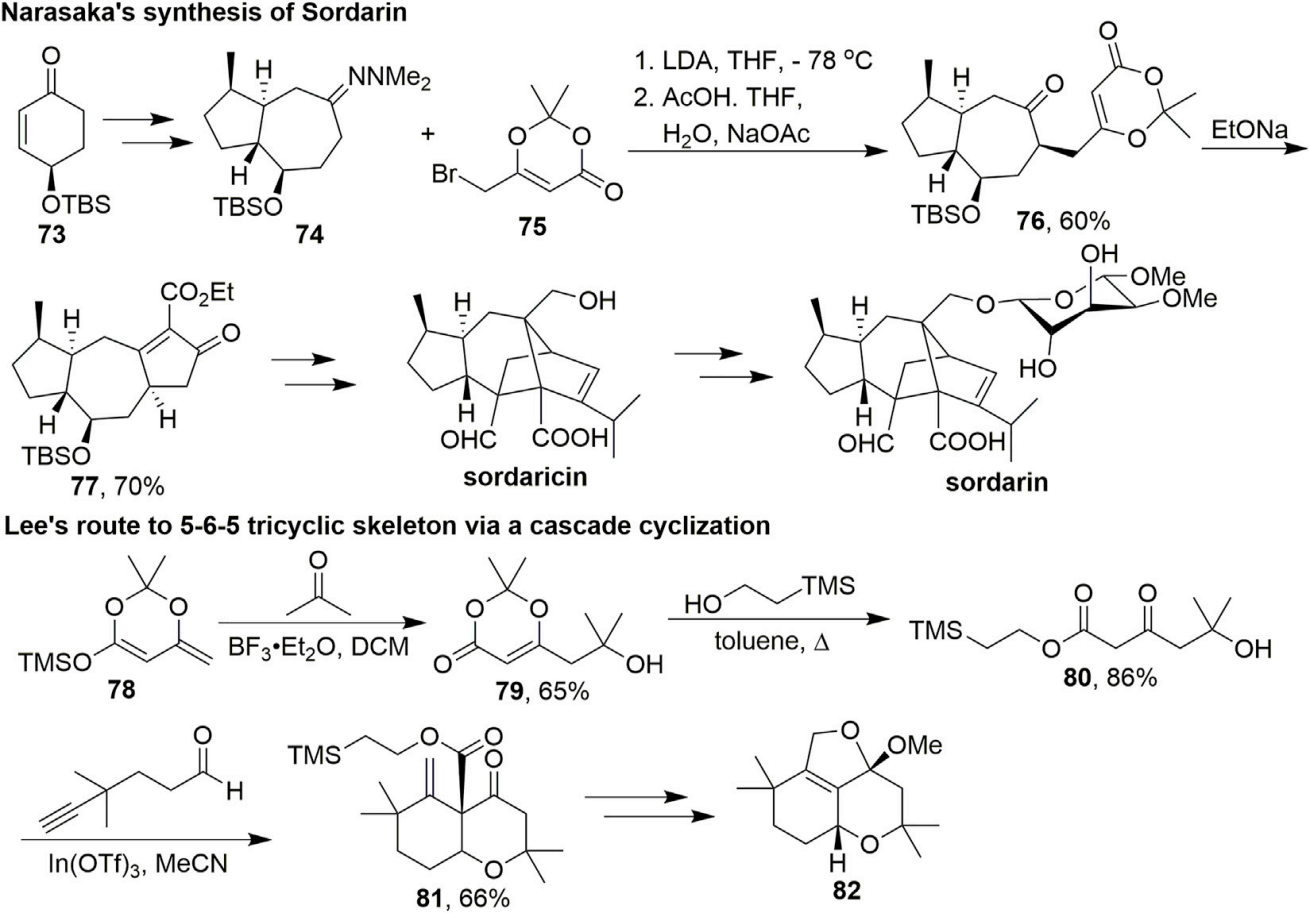
Applications of dioxinones in Narasaka and Lee’s studies.

In 2006, Narasaka et al. reported the asymmetric synthesis of sordarin and sordaricin (Scheme 7, Chiba et al., 2006). Sordarin has a tetracyclic cage-like structure with a glycosidic moiety which makes it particularly challenging to synthesize. They started with optically active **73**, which could prepare **74** in five steps. Dioxinone **75** was used as an alkylation reagent and added dropwise to a mixture of **74** and LDA. After cleavage of *N*,*N*-dimethylhydrazone, the resulting ketone **76** was treated with sodium ethoxide in ethanol to give tricyclic keto ester **77** *via* deprotection of the acetonide group and subsequent condensation. Sordaricin was then obtained through multistep transformations, and glycosylation of sordaricin completed the synthesis of sordarin.

In 2011, Lee’s group developed a novel route to construct the 5-6-5 tricyclic furanochroman skeleton of phomactin A *via* a Prins/Conia-ene cascade cyclisation (Scheme 7, Huang eta al., 2011). Phomactins represent a new class of platelet-activating factor (PAF) antagonists isolated from the marine fungus *Phoma* sp. and inhibit PAF-induced platelet aggregation (Sugano et al., 1991, 1996). Dioxinone **79** was obtained *via* a vinylogous aldol reaction between **78** and acetone. The product was then heated in toluene with the appropriate alcohol to give β-ketoester **80**. Subsequently, a variety of factors of this cascade approach, including Lewis acids, solvents, and temperature, were examined. This multifunctional intermediate successfully underwent a Prins/Conia-ene cascade cyclisation with the alkynaldehyde facilitated by In(OTf)_3_ to give **81** in 66% yield. The desired tricyclic skeleton was obtained after several subsequent steps.

**SCHEME 8:**
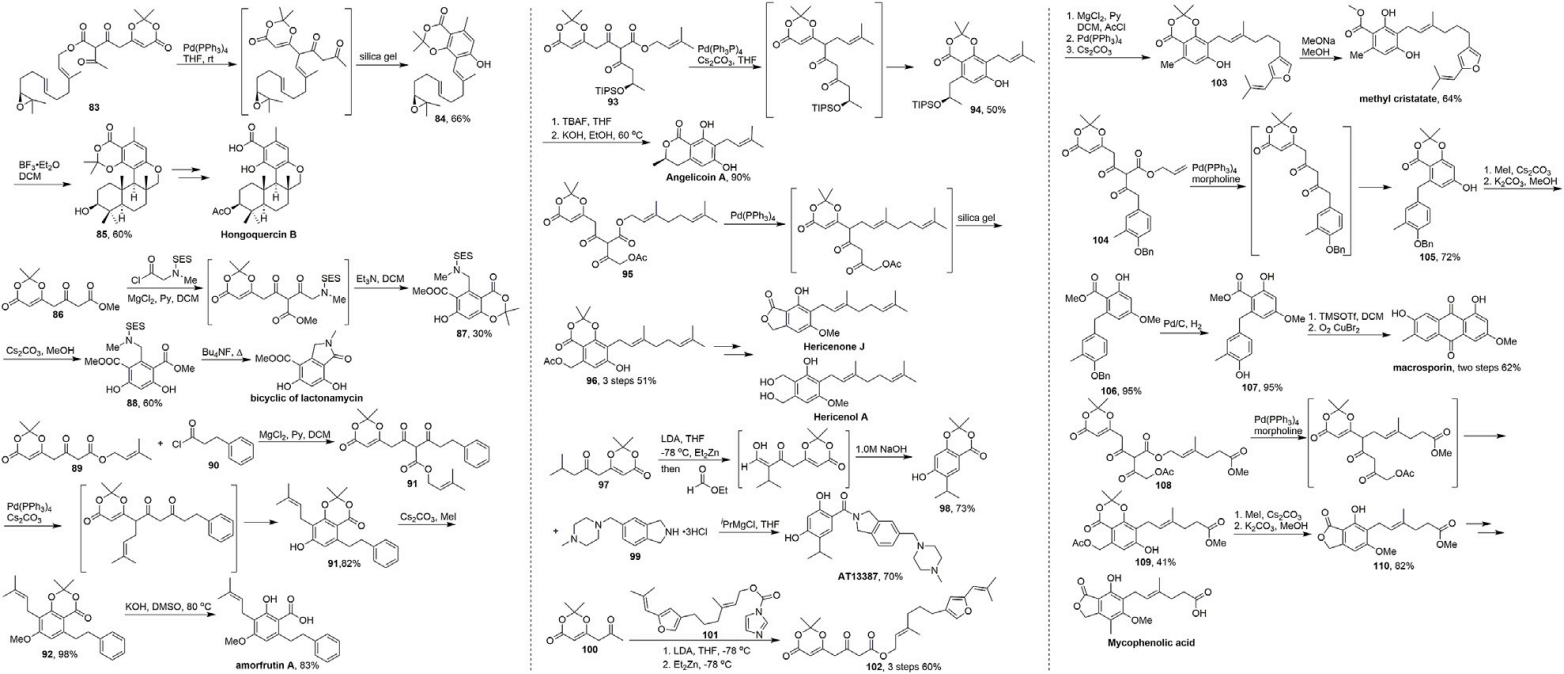
Barrett’s synthesis of terpene resorcylates and dihydroxy-isoindolinone derivatives.

In 2014, Barrett and Barrett (2014) reported the total synthesis of the complex natural product hongoquercin B, which has been isolated from extracts of an unidentified terrestrial fungus and exhibits antibiotic activity against vancomycin-resistant *Enterococcus faecium* and methicillin-resistant *Staphylococcus aureus* (Scheme 8, Roll et al., 1998; Ma et al., 2018). The key advanced intermediate **84** was formed *via* a decarboxylation allylation tandem aromatisation of **83**. Treatment of ester **83** with Pd(PPh_3_)_4_ at room temperature gave a diketo-dioxinone intermediate, which was readily aromatised over silica gel to give resorcylate **84** in 66% yield over three steps. Notably, only the desired linear *E*,*E*-isomer was obtained in this sequence. Subsequent addition of BF_3_·Et_2_O in dichloromethane furnished pentacyclic skeleton **85** in 60% yield by cascade cyclisation. The total synthesis of hongoquercin B was completed in two steps through functional group modifications.

Since the first example reported by Barrett in 2011, more than ten molecules have been synthesized using this approach *via* the cascade cyclisation-aromatization, which is an efficient and concise method for constructing dioxinone-resorcylate especially the terpene resorcylates and dihydroxy-isoindolinone derivatives. Barrett’s works (Scheme 8) were carried out using dioxinone derivatives as an important synthetic building block to achieve the diketo-dioxinone ester, palladium catalyzed migratory, decarboxylative prenylation–aromatization sequence as the key cascade process to establish the core frameworks of natural products with the mild conditions, easy work-up, wide scopes and high yield.

In 2011, they developed a concise five-step synthesis to the E, F-ring system of lactonamycin (Jacques et al., 2011). Dioxinone ketoester **89** and chloride **90** were used to provide the key in intermediate. Then subsequent reaction of diketoester–dioxinone **91** with Pd(PPh_3_)_4_ and cesium carbonate resulted in decarboxylative prenyl migration and formation of the resorcylate **92**. Amorfrutin A was obtained after the phenol methylation and saponification (Laclef et al., 2012). In 2012, Barrett and coworkers used different dioxinone-diketoesters **93** and **95** to provide the corresponding products **94** and **96** of the tandem process in good yields over several steps. Finally, they accomplished three terpene resorcylates angelicoin A, hericenone J and hericenol A in just five steps (Cordes et al., 2012). In 2012, Patel and Barrett (2012) completed the total synthesis of a Hsp90 nnhibitor AT13387. They started with ketoester **97** and gave the dioxinone-resorcylate **98** in 73% yield and obtained AT13387 after the saponification and condensation in total 3 steps from **97**. Barrett’s group finished the total synthesis of macrosporin (Cordes and Barrett, 2013), mycophenolic acid (Brookes et al., 2013) and methyl cristatate (George et al., 2013) utilizing this tandem strategy in 2013. In general, this conversion strategy has great advantages in the synthesis of natural products containing aryl-phenol structures and provides a new convenient route for this kind of natural product.

**SCHEME 9:**
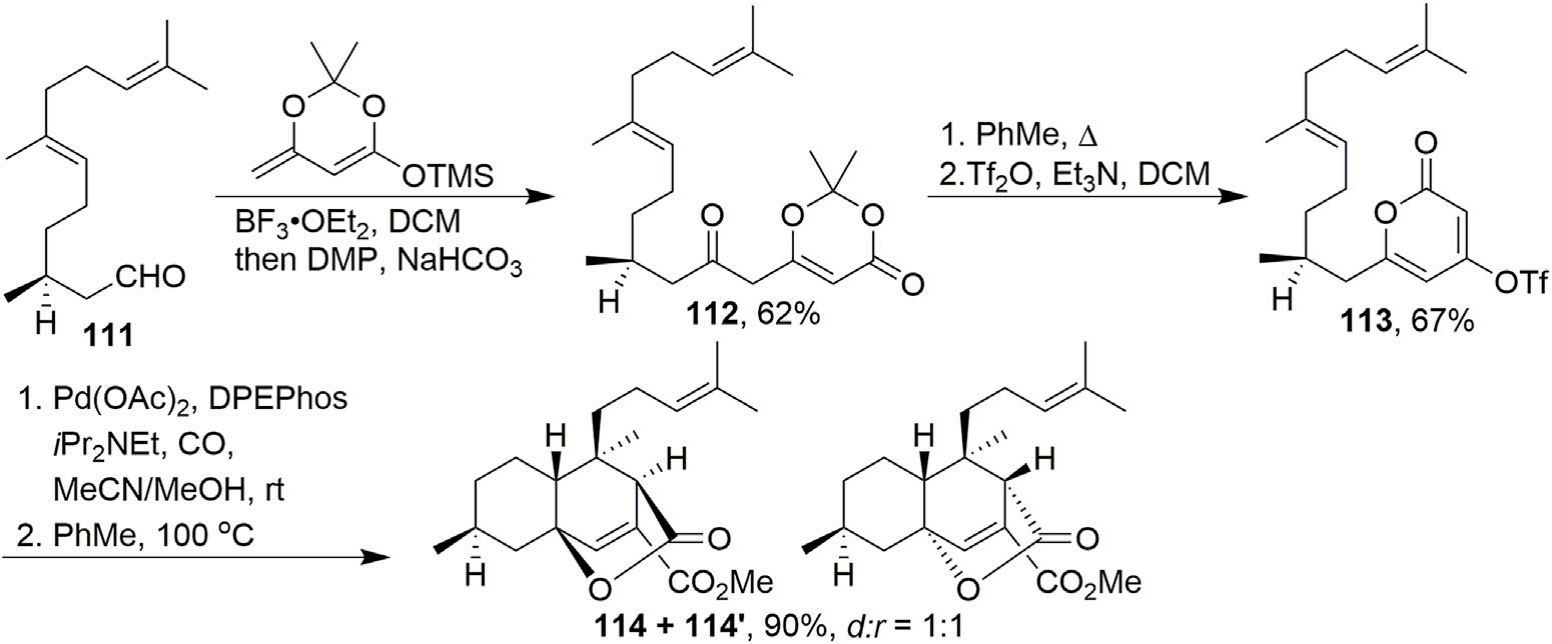
Maimone’s protocol to synthesise the tricyclic skeleton of chatancin.

In 2015, Zhao and Maimone (2015) used dioxinone as a key building block to construct key intermediate **114** in the asymmetric total synthesis of chatancin (Scheme 9). A vinylogous adol reaction was employed to convert aldehyde **111** and an enol ether into a secondary alcohol, followed by treatment with DMP to furnish dioxinone **112**. After heating in toluene and the intramolecular capture of acylketenes, treatment of the product with Tf_2_O successfully gave **113**. Pd-catalysed carbonylation of **113** dissolved in a mixture of acetonitrile and methanol successfully provided desired product **114**. It was discovered that heating a toluene solution of the ester for 4 days smoothly elicited a [4 + 2] cycloaddition in high yield. This process forged four stereocentres in a single operation. Equimolar amounts of diastereomers **114** and **114′** were formed during this process.

## New synthetic methods research

Since early efforts on the synthesis of perhydrohistrionicotoxin in 1989, using dioxinone to build the β-keto acid derivatives used to access macrocyclic and terpenoid natural products has been investigated in many groups around the world. In this section, select recent examples illustrate contemporary methodologies for applications involving dioxinone derivatives.

**SCHEME 10:**
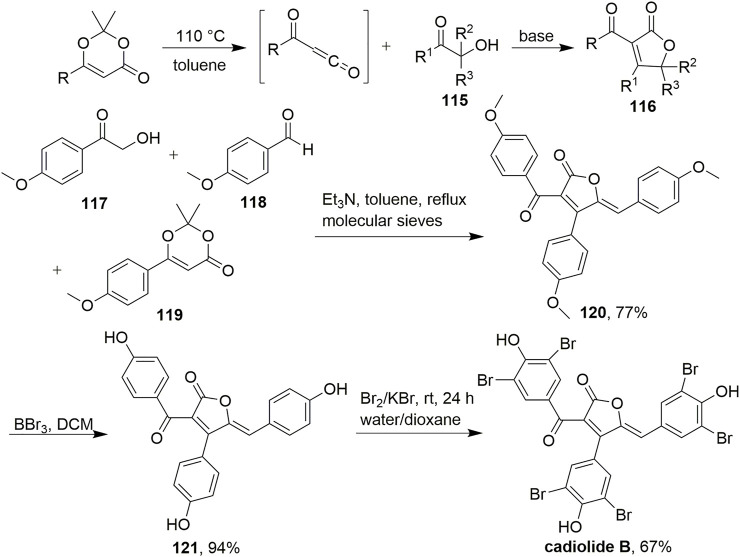
A new cycloaddition to prepare novel multisubstituted γ-butyrolactones.

In 2013, a new cycloaddition methodology to synthesise novel multisubstituted γ-butyrolactones was developed by Leleu et al. (Scheme 10, Peixoto et al., 2013). In this study, multisubstituted γ-butyrolactones **116** were prepared in a one-step procedure by capturing the thermal fragmentation of dioxinones in the presence of hydroxy ketones **115** under basic conditions. Various parameters were considered for the one-step synthesis of γ-butyrolactones, including the amount of base or dioxinone and the nature of the base. Ultimately, the use of 0.5 equiv. triethylamine and 1.5 equiv. dioxinone resulted in the highest yields. Under these conditions, acylfuranone **120** was prepared in one step from **117**, **118**, and **119** in 77% yield. This method was extended to the one-pot multicomponent synthesis of densely functionalized γ-butyrolactones. This diversity-oriented approach provided expeditious access to various small-ring compounds with potentially high antimicrobial activities under mild conditions with easy handling procedures and a wide scope of substrates. This will most certainly find a broad range of applications in medicinal chemistry.

**SCHEME 11:**
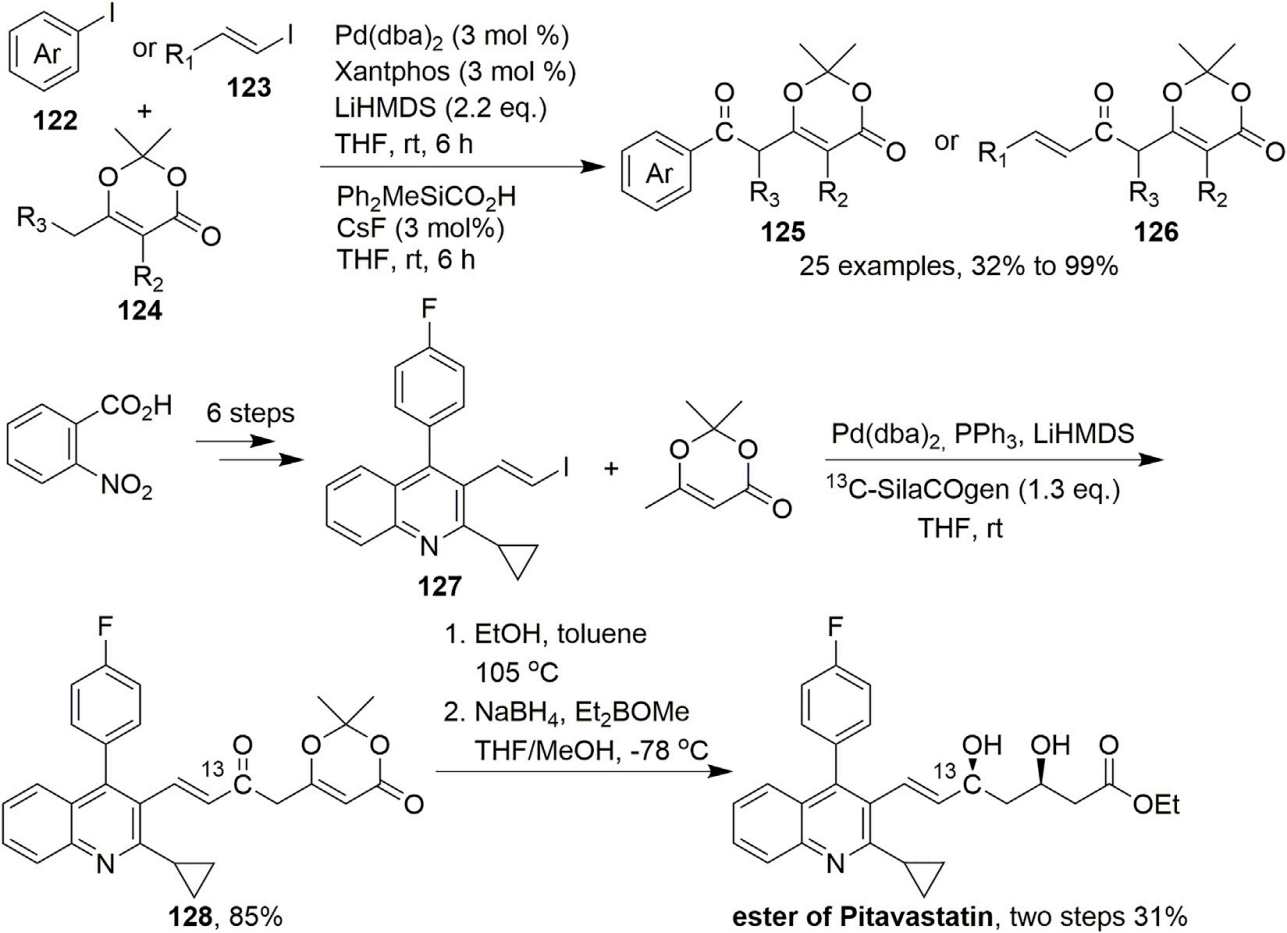
Pd-catalysed carbonylative couplings.

The palladium-catalysed arylation or vinylation of enolisable carbonyl and related compounds represents a viable and useful C–C bond-forming reaction, that is, widely applied in synthetic organic chemistry. In 2015, Lindhardt et al. developed a Pd-catalysed carbonylative coupling of aryl and vinyl halides with vinylogous enolates in which the C–C bond is formed exclusively at the γ-position (Scheme 11, Makarov et al., 2015). In this reaction, the conditions for the carbonylative coupling, including ligand, base, and solvent, were screened under Pd(dba)_2_ catalysis. Ultimately, using Xantphos as the ligand and LiHMDS as the base, this reaction gave an 82% yield under 3 mol% Pd(dba)_2_ catalysis in THF. The reaction was performed under mild conditions with various dioxinones and substituted aryl and alkenyl iodides to give aryl and alkenyl ketones **125** and **126**, respectively. Dioxinone was coupled to a range of aryl and vinyl iodides to provide 3,5-dicarbonyl acids with complete γ-selectivity. Furthermore, the carbonylation reactions were performed at room temperature with stoichiometric amounts of carbon monoxide. To apply this reaction to natural product synthesis, first, substituted quinoline **127** was readily prepared from 2-nitrobenzoic acid in six steps. Subsequently, ^13^C-SilaCOgen produced ^13^C-labeled dioxinone **128** according to the standard conditions for carbonylative coupling in an excellent isolated yield of 85%. Dioxinone opening was then accomplished using ethanol in toluene, which was followed by *syn*-diastereoselective reduction of the two ketones, ultimately affording the ^13^C-labeled ethyl ester of (±)-pitavastatin in 31% isolated yield over two steps. The synthesis of this corresponding ^13^C-labeled product was to indicated that this carbonylative coupling reaction could be used to synthesize drug molecules and also demonstrate the origin of carbonyl groups.

**SCHEME 12:**
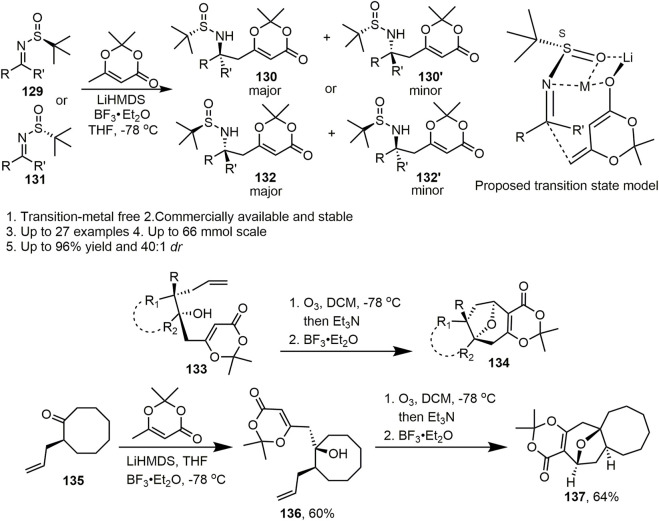
The application of dioxinone in Zhang’s work.

In 2017, Zhang et al. reported the full details of a general and practical diastereoselective approach towards the synthesis of δ-amino acid derivatives by vinylogous Mannich reactions between *N*-*tert*-butanesulfinyl imines and dioxinone lithium dienolate (Scheme 12, Li et al., 2017). In this study, systematic screening of the reaction conditions, especially the base and Lewis acid, was conducted to optimise the yield and diastereoselectivity of this reaction. It was clear that the base and Lewis acid had a major influence on the reaction. Notably, the corresponding product (**130**, **130′** and **132**, **132’**) was isolated in up to 87% yield and 40:1 d.r. in the presence of 2 equiv. BF_3_·Et_2_O. With the scope of the diastereoselective vinylogous Mannich reactions having been investigated, a variety of aryl, alkyl, and cyclic substituted *N*-*tert*-butanesulfinyl aldimines and ketimines (**129** and **131**) were obtained in mild to excellent diastereoselectivities (d.r. 1.2:1 to >40:1) and yields (20%–96%) under optimised conditions. Moreover, heterocyclic- and fused-ring–substituted imines gave moderate to excellent yields and diastereoselectivities. In the proposed transition state model, the author inferred that the imine was activated by coordination with BF_3_, and Si-face addition of the dioxinone-derived lithium dienolate led to major products with S-configuration for the newly formed stereocentre. This reaction provides a novel method for the synthesis of amino acids and chiral amines. Most importantly, the corresponding products can undergo many valuable transformations. Additionally, this method provides a foundation for the synthesis of natural products.

Two years later, Zhang’s group developed a practical method for the construction of an oxa-bridged bicyclic ring system *via* an oxidative-cleavage/Prins-cyclisation approach towards the synthesis of highly functional oxa-bridged seven-, eight-, and nine-membered rings (Scheme 12, Wang et al., 2019). Zhang et al. used various substituted aldehydes, ketones, and Weinreb amides as starting materials. Alcohol **133** was successfully obtained in two steps and set the stage for the proposed oxidative cleavage and Prins cyclisation. In the initial studies, oxidation by ozone provided a semiketal that formed an oxonium ion upon treatment with BF_3_·Et_2_O, and cyclisation gave the desired oxa-bridged compound. According to the established route, Zhang et al. extended the general utility of this process to synthesise ring systems of other sizes, including 7/8/9-membered oxa-bridged rings. Substrates bearing cyclopentane, cyclohexane cycloheptane, and cyclooctane also reacted well and provided tetracyclic products. In addition, seven−eight fused, eight−eight fused, and nine-membered ring systems could be constructed using this methodology. Notably, **137** can also be obtained from ketone **135**, which is the core skeleton of neoabyssomicin D. This straightforward method for the synthesis of oxa-bridged bicyclic ring systems in many natural products is flexible and enables the entry of virous of highly functionalized fused carbocycles. Reactions are easy to handle, highly diastereoselective, and can be performed on the Gram scale. This process is applicable for the synthesis of natural products containing an oxa-bridged bicyclic core skeleton.

## Conclusion

This review illustrates recent advances in the application of dioxinone derivatives to macrocyclic natural products, terpenoid synthesis, and new synthetic methods. Dioxinone derivatives have quickly become powerful, fascinating, and highly efficient tools in organic synthesis. Many researchers have contributed innovative and often practical methods that have established dioxinone derivatives as extremely versatile reagents for the robust and general synthesis of diverse classes of functional compounds, especially β-keto acid derivatives, even in complex natural products. The commercial availability in large quantities at low cost, the robustness and generality of its methods, and the prominence of macrocyclic and terpenoid natural products ensures that dioxinone-based strategies will continue to be some of the most extensively used methods in synthesis. Despite these achievements, new strategies and novel methodologies are both required and expected to facilitate the synthesis of complex molecules. However, due to the sensitivity of the dioxinone derivatives to strong base and high temperature, the application in synthesis is restricted to a certain extent. It is necessary to develop more novel dioxinone derivatives with diverse structure to make up for its defects and adapt to more extensive reaction conditions. We hope that organic chemists will continue to utilise dioxinone derivatives in their endeavours and make use of these multifunctional intermediates in the synthesis of even more complex natural products.

## Glossary

Ac: Acetyl
Ar: Aryl
Bn: Benzyl
Boc: *tert*-Butyloxycarbonyl
Bu: Butyl
Bz: Benzoyl
Cbz: Carbobenzoxyl
DBU: 1,8-Diazabicyclo[5,4,0]undec-7-ene
DCC: *N*,*N′*-Dicyclohexylcarbodiimide
DCE: Dichloroethane
DCM: Dichloromethane
DDQ: 2,3-Dicyano-5,6-dichlorobenzoquinone
DIBAL-H: Diisobutyl aluminium hydride
DIPEA: *N*,*N*-Diisopropylethylamine
DMAP: 4-Dimethylaminopyridine
DMF: *N*,*N*-Dimethylformamide
DMP: Dess-Martin Periodinane
DMSO: Dimethyl sulfoxide
dr: Diasetereomer ratio
EA: Ethyl acetate
ee: Enantiomeric excess
eq.: Equivalent
Et: Ethyl
IBX: 2-Iodoxybenzoic Acid
im: Imidazole
*i*-Pr: isopropyl
KHMDS: Potassium Hexamethyldisilazide
LDA: Lithium diisopropylamide
LHMDS: Lithium Hexamethyldisilazide
*m*-CPBA: *meta*-Chloroperbenzoic Acid
Me: Methyl
MOM: methoxymethyl acetal
PCC: Pyridinium chlorochromate
PE: Petroleum ether
Ph: phenyl
PMB: *p*-methoxybenzyl
PMBCl: *para*-Methoxybenzyl
PPTS: Pyridinium toluene-4-sulphonate
*p*-TsOH: *p*-Toluenesulfonic acid
Py: pyridine
rt: Room temperature
TBAF: Tetrabutylammonium fluoride
TBS: *tert*-Butyldimethylsilyl
TBDPS: *tert*-Butyldiphenylchlorosilyl
TEA: Triethylamine
Tf: trifluoromethanesulfonate
TFA: Trifluoroacetic acid
TFAA: trifluoroacetic anhydride
THF: Tetrahydrofuran
TLC: Thin layer chromatography
TIPS: Triisopropylsilyl
TMS: Trimethylsilyl
Ts: *p*-toluenesulfonyl
